# Angiographic features and transarterial embolization of retained placenta with abnormal vaginal bleeding

**DOI:** 10.1186/s42155-021-00265-z

**Published:** 2021-11-02

**Authors:** Ryo Takaji, Hiro Kiyosue, Miyuki Maruno, Norio Hongo, Ryuichi Shimada, Satomi Ide, Kohei Tokuyama, Mamiko Okamoto, Yasushi Kawano, Yoshiki Asayama

**Affiliations:** 1 Departments of Radiology, Oita University Faculty of Medicine, Yufu City, Oita 879-5593 Japan; 2 Department of Obstetrics and Gynecology, Oita University Faculty of Medicine, Yufu, Oita 879-5593 Japan

**Keywords:** Retained placenta, Angiography, Transarterial embolization

## Abstract

**Objectives:**

To clarify characteristic angiographic features and clinical efficacy of selective transarterial embolization (TAE) of retained placenta with abnormal vaginal bleeding.

**Methods:**

The study cohort comprised 22 patients (mean age, 33.5 years; range, 22–24 years) who underwent selective TAE for retained placenta with abnormal bleeding between January 2018 and December 2020 at our institution. Angiographic images were reviewed by two certified radiologists with consensus. Medical records were reviewed to evaluate the efficacy of TAE. Angiographic features of retained placenta, technical success (disappearance of abnormal findings on angiography), complications, clinical outcomes (hemostatic effects and recurrent bleeding) were evaluated.

**Results:**

Pelvic angiography showed a dilated vascular channel mimicking arteriovenous fistulas or an aneurysm contiguous with dilated uterine arteries in the mid-arterial–capillary phase in 20 patients; it showed contrast brush in the remaining two patients. TAE technical success was achieved in all patients. No major complications were observed in any patients. Fifteen patients were followed up with expectant management after TAE; all but one patient showed no re-bleeding during the follow-up period (mean follow-up interval, 3.4 months; range, 1–17 months). One patient showed minor rebleeding, which resolved spontaneously. Seven patients underwent scheduled hysteroscopic resection within 1 week after TAE, and no excessive bleeding was observed during or after the surgical procedure in all seven patients.

**Conclusions:**

The characteristic angiographic feature of retained placenta is “dilated vascular channel that mimic low flow AVM.” TAE is a safe and effective treatment to manage retained placenta with abnormal bleeding.

## Introduction

Retained placenta is a major cause of postpartum hemorrhage. Notably, retained placenta in the presence of severe postpartum hemorrhage has a reported frequency of 11.4%–33.3% among the patients with severe postpartum hemorrhage (Nyfløt et al. 2017; Hulse et al. 2020; Kodan et al. 2020; Shams et al. 2020; Sosa et al. 2009; Widmer et al. 2020). Retained placenta can cause life-threatening severe vaginal bleeding. Hence, diagnosis and appropriate management of retained placenta are important.

Recently, transarterial embolization (TAE) has been established as a treatment option for uncontrollable postpartum hemorrhage (Tourne et al. 2003; Soyer et al. 2011). Based on a case series study and corresponding literature review, Chauleur et al. found that uterine artery embolization was safe and effective for postpartum hemorrhage caused by placenta accreta (Chauleur et al. 2008). To our knowledge, few studies (Bazeries et al. 2017; Kimura et al. 2020; Jiang et al. 2020; Takeda and Koike 2017) specifically report the use of TAE for treatment of retained placenta, although the use of TAE in postpartum hemorrhage is well described as a safe and effective treatment option. Furthermore, characteristic angiographic features of retained placenta have not yet been elucidated although successful embolization requires recognition of the target lesion on angiography.

The objectives of this study were to clarify characteristic angiographic features of retained placenta with vaginal bleeding and to evaluate the efficacy and safety of selective TAE for management of abnormal vaginal bleeding specifically caused by retained placenta.

## Materials and methods

### Patient selection

This retrospective study was approved by the ethics committee of our institution, and the requirement for informed consent was waived because of the retrospective nature of the study. The radiology databases and electronic medical records of our institution were reviewed to identify patients with retained placenta who had abnormal vaginal bleeding. Between January 2018 and December 2020, 22 consecutive patients were retrospectively extracted based on the following criteria: (a) had undergone selective TAE; (b) had a symptomatic vaginal bleeding; (c) clinically diagnosed retained placenta based on the patient’s medical history, clinical examinations, and transvaginal ultrasonographic (TVUS) findings. (d) had no other endometrial diseases. Retained placenta tissue with marked vascularity was confirmed by TVUS in all patients. In addition, biphasic contrast-enhanced (CE) CT (21 patients) or dynamic CE MRI (one patient) was performed to evaluate the location of the retained placenta and its potential feeding arteries.

The characteristics of the 22 patients in this study are summarized in Table 1. The mean patient age was 33.5 years (range, 22–44 years). Of these 22 patients, 17 had a history of dilation and curettage (three patients had spontaneous miscarriage and 14 patients underwent termination of pregnancy). Of the remaining five patients, three underwent vaginal delivery and two underwent cesarean resection. The mean gestational ages in the abortion and delivery groups were 9.3 weeks (range, 6–19 weeks) and 29.4 weeks (range, 16–41 weeks), respectively. Three of the 22 patients had severe vaginal bleeding, and 19 patients had continuous minor vaginal bleeding. The mean size of retained placenta on CE-CT or MRI was 20.9 mm (range, 5–55 mm). The mean interval from abortion or delivery to TAE was 44.3 days (range, 11–100 days). The mean hemoglobin level of the 22 patients was 12.4 g/dL (range, 6–14.7). Two patients (9%) received blood transfusion before TAE procedure (4, 6 unit). Majority of the patients (20/22, 91%) had undergone elective TAE and emergency TAE was performed in 9% (2/22).

**Table 1:** Characteristics of 22 women with vaginal hemorrhage due to retained placenta

Characteristics	*n* = 22
Age [years; mean (range)]	33.5 (22–44)
Gravidity [n; mean (range)]	2.7 (1–6)
Parity [n; mean (range)]	0.9 (0–2)
Abortion [n]	
Spontaneous miscarriage and dilation and curettage	3
Termination of pregnancy by dilation and curettage	14
Delivery mode [n]	
Vaginal delivery	3
Cesarean delivery	2
Gestational weeks	
Abortion [weeks; mean (range)]	9.3 (6–19)
Delivery [weeks; mean (range)]	29.4 (16–41)
Hemorrhagic event [n]	
Significant bleeding	3
Continuous small amount of bleeding	19
Hemoglobin* [g/dL; mean (range)]	14.7 (6–14.7)
Size of retained placenta on CE-CT or MRI [mm; mean (range)]	20.9 (5–55)
Interval from abortion or delivery to TAE [days; mean (range)]	44.3 (11–100)

*CE-CT* contrast-enhanced CT, *TAE* transarterial embolization

*Red blood cell transfusion was performed in two patients (4, 6 unit)

### Angiography and selective transarterial embolization

The decision indication for the procedure was taken by multidisciplinary agreement referring to TVUS and CE-CT or MRI findings. All TAE procedures were performed by experienced interventional radiologists with more than 10 years of experience using digital subtraction angiography equipment (Infinix Celeve-I INFX8000C, Canon Medical Systems). Bilateral internal iliac angiographies with anterior and anterior oblique projections were performed using a 4-Fr or 5-Fr diagnostic catheter with injection of a nonionic iodinated contrast media (iopamidol, Iopamiron 350; Bayer Health Care) at a flow rate of 3–5 mL/sec (total volume, 9–15 mL) through an automatic injector. Then, a 2.7-Fr microcatheter was introduced through the diagnostic catheter into the ipsilateral uterine artery, and selective angiography of the uterine artery was subsequently performed with manual injection of 2–4 mL of contrast media. A 1.6-Fr or 1.9-Fr microcatheter was then advanced distally through the 2.7-Fr microcatheter to the target feeder when uterine angiography showed abnormal findings indicative of retained placenta and/or possible source of bleeding. We adopted triaxial system (1.6-Fr or 1.9-Fr non-taper microcatheter, 2.7-Fr microcatheter, 4-Fr or 5-Fr diagnostic catheter) to advance the microcatheter into the tortuous uterine artery. In this system it was easier to advance the 1.6-Fr or 1.9-Fr microcatheter because the 2.7-Fr microcatheter stabilized the position and prevented sagging or jumping. If uterine angiography did not show definitive findings of retained placenta, selective catheterization and angiography of the contralateral uterine artery were performed. Furthermore, target embolization was performed using gelatin sponge pieces or a mixture of n-butyl-cyanoacrylate (NBCA) and lipiodol (ratio of 1:3–5) when the 1.6-Fr or 1.9-Fr microcatheter was able to reach the appropriate feeders to the target lesion. If selective catheterization failed or numerous feeders originated from the proximal portion of the uterine artery, empirical embolization was performed with gelatin sponge pieces at the proximal site of the uterine artery. Embolic materials were selected by each operator in accordance with microcatheter reachability and angiographic features including the size and numbers of feeding arteries, as well as the size of the target lesion. After embolization, disappearance of the target lesion was confirmed by selective angiography of the bilateral uterine arteries and the bilateral internal iliac arteries. When residual supply to the target lesion was observed on contralateral angiography, embolization from the feeders of the contralateral uterine artery was performed by the same techniques. TAE technical success was defined as disappearance of the target lesion during the final angiography examination.

### Imaging interpretation

Two experienced radiologists reviewed all images obtained before TAE treatment in all 22 patients. They evaluated whether the angiographic findings corresponded to retained placenta in each patient. Coronal maximum intensity projection (MIP) images of CE-CT or MRI in the arterial phase were used as a reference to identify retained placenta.

### Therapeutic decision after TAE procedure

The need for each patient to undergo additional hysteroscopic resection after TAE was assessed by the attending gynecologist. Although patients were scheduled for hysteroscopic resection after TAE during the early portion of the study period, conservative management was preferred during the late portion of the study period. Expectant management after TAE was performed in 15 patients (expectant management group); hysteroscopic resection was performed within 1 week after the TAE procedure in the remaining seven patients (surgical management group). Clinical success was defined as the absence of re-bleeding requiring additional treatment in the expectant management group; it was defined as surgical completion without excessive intraoperative bleeding (≥2000 mL) in the surgical management group. Clinical success was investigated by reviewing medical and operative records. In the expectant management group, vascularity of retained placenta on TVUS or CE-CT within 1 week and more than 1 month after the TAE procedure were also evaluated.

## Results

The angiographic findings and results of selective TAE are summarized in Table 2.

**Table 2:** Angiographic findings and TAE procedures

	n = 22
Angiographic findings [n (%)]	
Contrast blush	2 (9%)
Dilated vascular channel that mimic low flow AVM*	20 (91%)
Pseudoaneurysm	0 (0%)
Extravasation	0 (0%)
Embolization site [n (%)]	
Target embolization	15 (68%)
Empirical embolization**	7 (32%)
Embolic material [n (%)]	
GS	18 (82%)
NBCA	3 (14%)
GS and NBCA	1 (4%)
Technical success***	22 (100%)
Complication [n (%)]****	
Transient hypotension	2 (9%)
Gluing microcatheter into blood vessel	1 (4%)

* Dilated vascular channel contiguous with multiple feeders from dilated uterine arteries in the mid-arterial to capillary phase, which drained into the uterine veins in the capillary to venous phase

** Reasons for Emprical embolization were failed super selective catheterization into target feeders (*n* = 6) and large retained placenta with numerous feeders (*n* = 1)

***Technical success was defined as disappearance of the target lesion during the final angiography examination

****Serious complications were not observed (complications were classified as CIRSE grade 1)

*NBCA* N-butyl-cyano-acrylate, *GS* gelatin sponge

### Angiographic findings

**Fig. 1:**
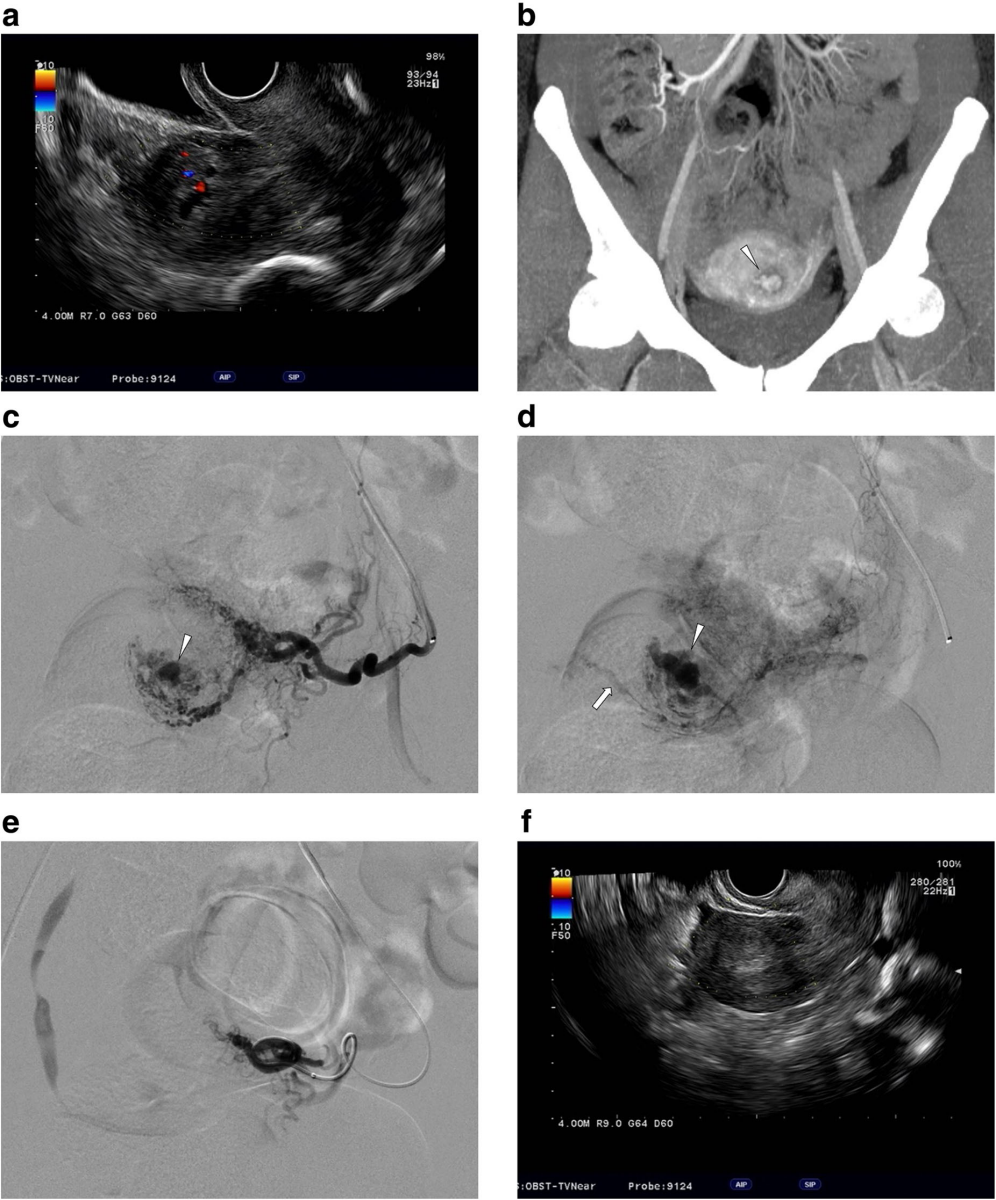
A 33-year-old woman with vaginal bleeding caused by retained placenta. a. Color doppler TVUS showed vascularity (arrowhead) in the uteroplacental tissue. b. Coronal MIP image of arterial phase CE-CT showed intrauterine vascular mass (arrowhead) fed by left uterine artery. c. Left uterine arteriogram showed dilated vascular channel (arrowhead) in arterial phase uterine arteriography. d. Venous drainage (arrow) from dilated vascular channel (arrowhead) depicted in the capillary phase. Drainage vein connected to right uterine vein in the venous phase. Microcatheter was inserted into the blood sinus feeding artery. Target embolization was performed using GS. e. Post-embolization left uterine arteriography confirmed disappearance of the dilated vascular channel. f. At 2 days after the TAE procedure, the intrauterine hyper vascular lesion disappeared on TVUS. In this case, expectant management after TAE was chosen and no re-bleeding was observed

**Fig. 2:**
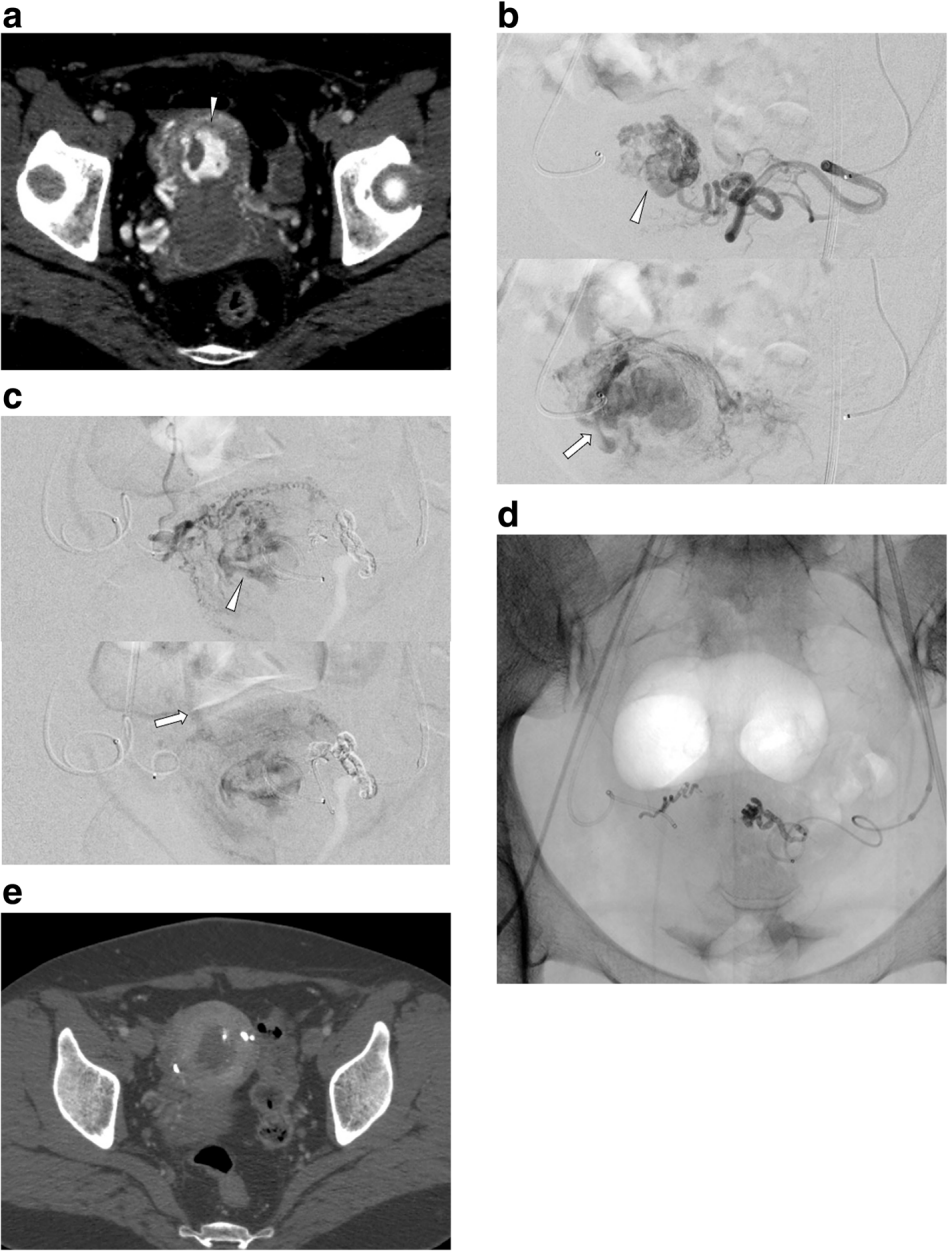
A 42-year-old woman with vaginal bleeding caused by retained placenta. a. Arterial phase CE-CT showed intrauterine enhancing lesion (arrowhead). b, c. Bilateral uterine arteriogram showed dilated vascular channel (arrowhead) in the arterial phase. Venous drainage (arrow) was depicted in the capillary phase through the vascular channel. d. Bilateral uterine arteries were embolized using 25% NBCA diluted with iodized oil. After the TAE procedure, expectant management was chosen and no re-bleeding was observed. e. At 1 month after the TAE procedure, the intrauterine enhancing lesion disappeared on CE-CT

Pelvic angiography (including internal iliac angiography and uterine angiography) showed a dilated vascular channel contiguous with multiple feeders from dilated uterine arteries in the mid-arterial to capillary phase, followed by drainage into the uterine veins in the capillary to venous phase in 20 patients (Figs. 1 and 2). Those angiographic features mimicked low-flow arteriovenous malformation/fistulas, but there were no early venous filling. In the remaining two patients, pelvic angiography showed contrast blush corresponded to a retained placenta in the mid-arterial to capillary phase.

### Results of selective TAE procedure

Regarding embolic materials, gelatin sponge (GS) pieces (1–2 mm) were used in 18 patients (Figs. 1), NBCA-lipiodol mixture in three patients (Fig. 2), and both of these materials in one patient. Target embolization was performed in 15 patients and empirical embolization was performed in the remaining seven patients. Reasons for empirical embolization were failed super selective catheterization into target feeders (*n* = 6) and large retained placenta with numerous feeders (*n* = 1).

Angiography after selective TAE showed disappearance of abnormal findings related to retained placenta in all 22 patients; therefore, the rate of TAE technical success was 100%, regardless of embolic materials or target/non-target embolization.

No major complications were observed. Three minor procedure-related complications (CIRSE grade 1) were observed: transient hypotension (*n* = 2) and gluing microcatheter in a feeder (*n* = 1). Transient hypotension resolved with conservative management. In the patient with an NBCA-adhered microcatheter, the amputated catheter tip remained in the left uterine artery.

### Clinical outcome

The clinical success rates in the expectant management and surgical management groups were 100% (15/15 patients) and 100% (7/7 patients), respectively. Tables 3 shows the TAE clinical outcomes in the expectant management group. A few days after TAE, vascularity of the retained placenta on TVUS or CE-CT was markedly reduced (*n* = 8) or disappeared (*n* = 7). Follow-up TVUS and/or CE-CT at 1 month after TAE showed no abnormal blood flow in the uterus in all 15 patients. Complete hemostasis without recurrent bleeding was achieved in 14 of these 15 patients. In one patient, minimal vaginal bleeding occurred after TAE, but spontaneously disappeared within 1 month.

**Table 3:** Clinical outcomes of TAE without hysteroscopic resection

Expectant management group (*n* = 15)	
Follow-up period [months; mean (range)]	3.4 (1–17)
Clinical success*	15 (100%)
Vascularity of retained placenta on TVUS or CE-CT [n (%)]	
Few days after TAE	
Marked reduction	8 (53%)
Disappearance	7 (47%)
More than 1 month after TAE	
Disappearance	15 (100%)
Vaginal bleeding after TAE [n (%)]	
No bleeding	14 (93%)
Minimal bleeding**	1 (7%)

*Clinical success was defined as the absence of re-bleeding requiring additional treatment in the conservative treatment group

** Minimal bleeding spontaneously disappeared within 1 month

*TVUS* transvaginal ultrasound, *CE-CT* contrast-enhanced CT, *TAE* transarterial embolization

Prevention effect of intraoperative bleeding after TAE are summarized in Table 4. In the surgical management group, scheduled hysteroscopic resection of retained placenta was performed within 1 week; no patients showed excessive bleeding during surgical procedures. All seven patients showed no recurrent vaginal bleeding after surgery.

**Table 4:** Prevention effect of intraoperative bleeding after TAE

Surgical management group (*n* = 7)	
Clinical success*	7 (100%)
Intraoperative bleeding [n (%)]	
Almost no bleeding	7 (100%)
Excessive bleeding	0 (0%)

*Clinical success was defined as surgical completion without excessive intraoperative bleeding (≥2000 mL) in the surgical management group

TAE, Transarterial embolization

## Discussion

**Fig. 3:**
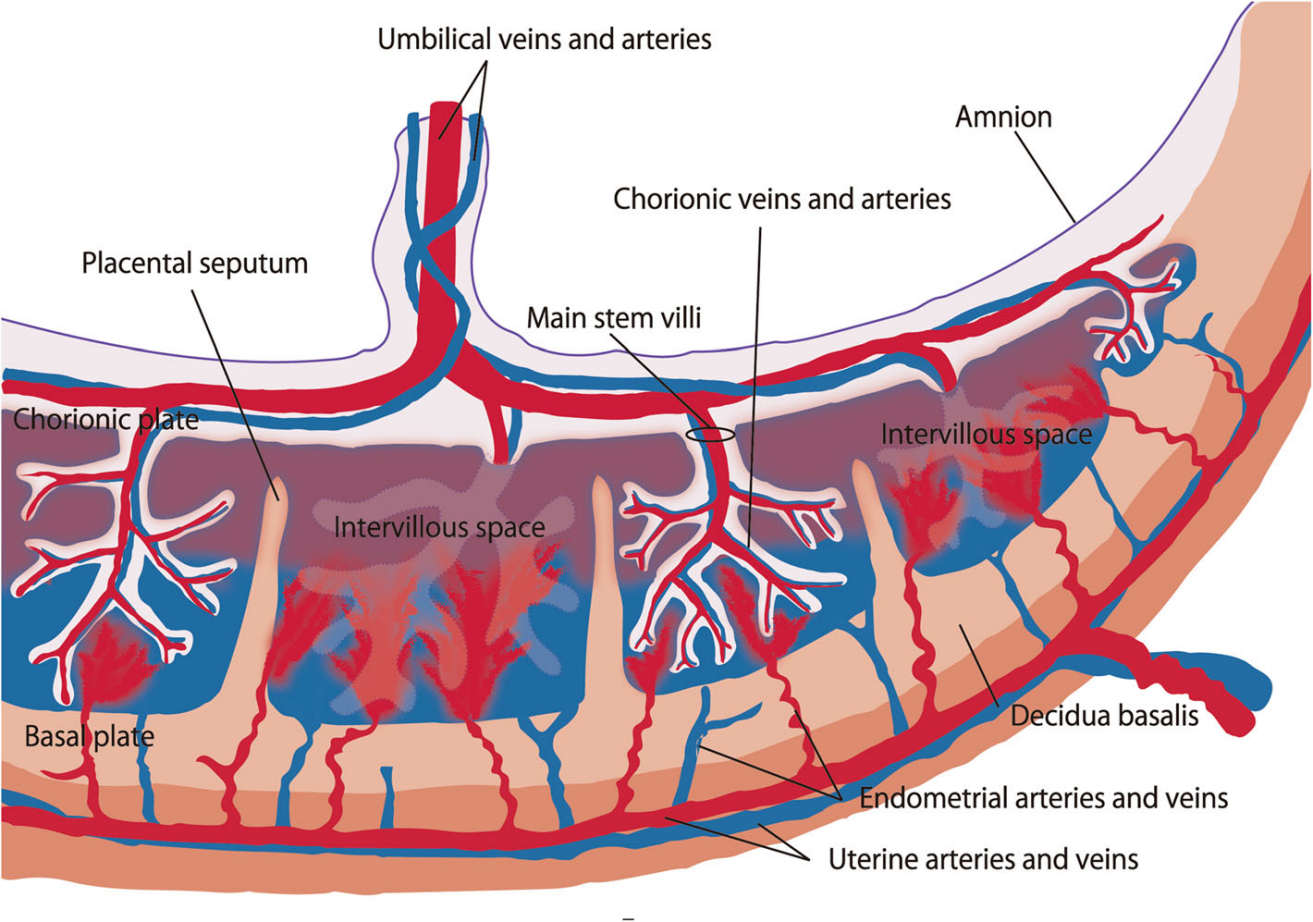
Schematic drawing of the placenta (cross-sectional image). The chorionic plate (fetal side) is a mass of connective tissue that contains the amnion, main stem villi, and chorionic arteries and veins. The basal plate (maternal side) consists of trophoblastic and decidual cells; it contains the placental septa, decidua basalis, and endometrial arteries and veins. The chorionic and basal plates are separated by the intervillous space; exchange between fetal and maternal circulatory systems occurs between the main stem villi and the maternal endometrial vessels in this space

**Fig. 4:**
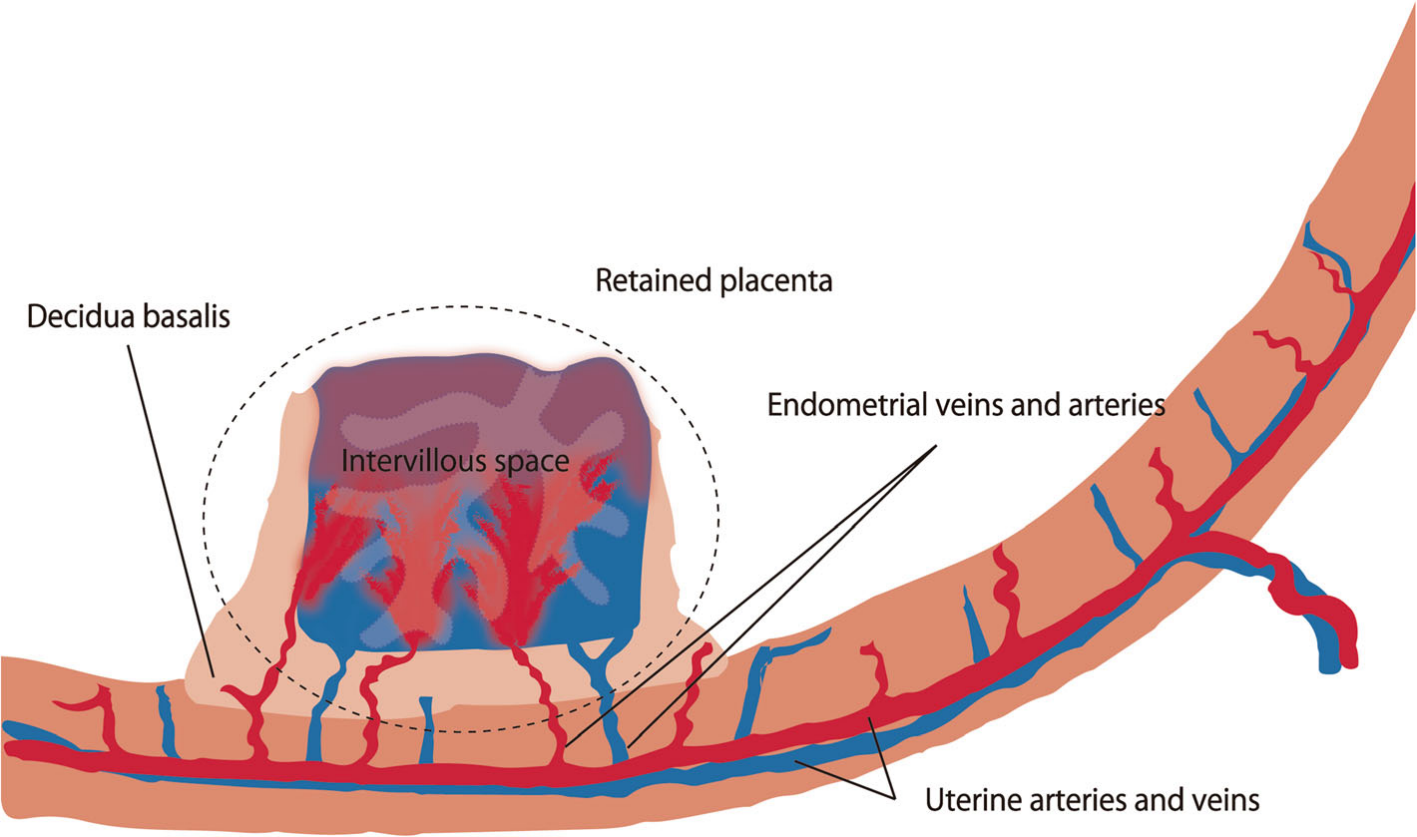
Schematic drawing of the retained placenta (cross-sectional image). The retained placenta may consist of intervillous space and decidua basalis. Endometrial arteries and veins, which are branches of uterine arteries and veins, are connected to each other through the intervillous space. Angiographic findings including dilated vascular channel and contrast blush may correspond to remnant intervillous space. Endometrial arteries and veins connecting to the intervillous space may represent low-flow AVM-like findings

Thus far, few reports have specifically mentioned angiographic features of retained placenta in patients with postpartum hemorrhage (Bazeries et al. 2017; Kimura et al. 2020; Kitahara et al. 2011]. In those reports, angiographic features of retained placenta have included tortuous dilated uterine artery flowing into a sac-like structure, intrauterine vascular lesion with or without arteriovenous (AV) shunt, focal contrast blush, and pseudoaneurysm. In the present study, most patients (91%) showed a characteristic finding of dilated vascular channel in the mid-arterial to capillary phase which mimics low-flow arteriovenous malformation/fistula. However, the vascular lesion drained into the uterine veins in the capillary to venous phase without early venous filling. The placenta consists of the chorionic and basal plates, and the intervillous space lies between these two plates. The main stem villi, consisting of chorionic veins and arteries, project into the intervillous space. Maternal endometrial arteries and veins penetrate the basal plate; exchange between fetal and maternal circulatory systems occurs between the main stem villi and the maternal endometrial vessels in the intervillous space (Jansen et al. 2020; Cunningham et al. 2013; Bernischke 1967; Kaufmann 1985) (Fig. 3). In addition, uterine arteries and veins are presumed to exhibit arteriovenous anastomosis separate from this intervillous short-circuit (James et al. 2017). The retained placenta consists of intervillous space and decidua basalis. In cases of retained placenta, various extents of remnant intervillous space and arteriovenous anastomosis of endometrial arteries/veins could remain in the uterine cavity (Fig. 4). The angiographic finding of dilated vascular channel may correspond to remnant intervillous space. Furthermore, endometrial arteries and veins connecting to the intervillous space may represent one or more low-flow AV shunts mimicking arteriovenous malformation (AVM)-like findings. Uterine AVM is rare, and it involves abnormal vascular channels in the endometrium or myometrium with early venous filling during the early arterial phase (Vijayakumar et al. 2013; Ghai et al. 2003; Timmerman et al. 2003). Retained placenta can be incorrectly diagnosed as AVM. However, angiography in our study showed venous drainage from the dilated vascular channel of retained placenta was evident in the capillary to venous phase. This angiographic finding of apparently delayed venous drainage may differentiate retained placenta from uterine AVM.

Regarding TAE specifically for the treatment of retained placenta with bleeding, few case series focused on retained placenta have been reported. Bazeries et al. (2017) reported that TAE technical and primary clinical successes, using mainly microspheres (size: 700–1200 μm), were achieved in 90.3% (27/31) and 74.2% (23/31) of their patients. Kimura et al. (2020) reported higher rates of TAE technical and clinical success using GS (93%, 13/14; 100%, 14/14). NBCA embolization of retained placenta increta was described in a case report; complete occlusion and cure was achieved with single embolization (Hamaguchi et al. 2003). Jiang et al. (2020) reported favorable prevention effect of intraoperating bleeding of TAE using GS followed by hysteroscopic resection (intraoperating blood loss; ≤100 ml, 90.3%, 28/31, 100-400 ml, 6.5%, 2/31, ≥400 ml, 1%, 1/31). Takeda and Koike (2017) reported that TAE/TACE (using GS with or without dactinomycin) were key intervention for uterus preserving treatment of retained placenta accrete with marked vascularity (devascularization of retained placenta and uterine preservation were achieved in all of 38 patients). In addition, there are some papers mentioned about the usefulness and safety of TAE using GS and/or PVA for postpartum hemorrhage from various causes including retained placenta (Ko et al. 2017; Pelage et al. 1999; Horng et al. 2011). In our study, TAE for retained placenta was performed using GS and/or NBCA, according to the operator’s preference, and favorable outcomes were achieved.

Retained placenta can spontaneously resolve with conservative management. Hence, asymptomatic patients with small and non-hypervascularized retained placenta may be candidates for conservative management (Takahashi et al. 2019; Jain and Fogata 2007; Lee et al. 2014). In this study, 15 patients underwent expectant management after TAE. Among these 15 patients, eight showed markedly reduced residual vascularity of retained placenta. Images collected at the 1-month follow-up showed vascular lesion disappearance in the uterus, and there were no cases of recurrent bleeding that required any treatment. The safety of uterine artery embolization has been indicated for resolution of post-partum hemorrhage (Chauleur et al. 2008). Common adverse effects related to the TAE procedure include high fever, acute pelvic inflammation, and hip pain (Chen et al. 2015; Liu et al. 2018). However, excessive embolization may cause serious complications, such as uterine necrosis and endometrial atrophy (Godfrey and Zbella 2001; Cottier et al. 2002). Furthermore, Ohmaru-Nakanishi et al. (2019) reported that patients who were treated for retained placenta with TAE were at risk of postpartum hemorrhage and difficulty in removing placenta in future pregnancies, although, there were no effect reproductive outcomes. As described previously, retained placenta has a regressive nature. Progressive occlusion of the blood sinus of retained placenta can occur after TAE. Therefore, excessive embolization should be avoided when the characteristic finding of retained placenta, dilated vascular channel that mimic low flow AVM, is identified during angiography examination. This information is important for interventional radiologists to determine the procedural endpoint of TAE for retained placenta with abnormal bleeding.

This study had several limitations including its retrospective nature, limited case number, and short follow-up period (mean, 3.4 months; range, 1–17 months). Larger prospective studies are needed to confirm the safety and efficacy of the TAE procedure as a monotherapeutic approach for retained placenta with abnormal bleeding.

In summary, the characteristic angiographic feature of retained placenta with vaginal bleeding is a dilated vascular chaneel fed by multiple uterine arterial branches in the arterial to capillary phase, which drains into the uterine vein in the capillary to venous phase. TAE using GS and/or NBCA can be a safe and effective treatment for management of abnormal bleeding caused by retained placenta.

## References

[CR1] Bazeries P, Paisant-Thouveny F, Yahya S, Bouvier A, Nedelcu C, Boussion F, Sentilhes L, Willoteaux S, Aubé C (2017). Uterine artery embolization for retained products of conception with marked vascularity: a safe and efficient first-line treatment. Cardiovasc Intervent Radiol.

[CR2] Bernischke K (1967). The pathology of the human placenta.

[CR3] Chauleur C, Fanget C, Tourne G, Levy R, Larchez C, Seffert P (2008). Serious primary post-partum hemorrhage, arterial embolization and future fertility: a retrospective study of 46 cases. Hum Reprod.

[CR4] Chen J, Chen W, Zhang L, Li K, Peng S, He M, Hu L (2015). Safety of ultrasound-guided ultrasound ablation for uterine fibroids and adenomyosis: a review of 9988 cases. Ultrason Sonochem.

[CR5] Cottier JP, Fignon A, Tranquart F, Herbreteau D (2002). Uterine necrosis after arterial embolization for postpartum hemorrhage. Obstet Gynecol.

[CR6] Cunningham FG, Leveno KJ, Bloom SL (2013). Implantation and placental development. Williams obstetrics.

[CR7] Ghai S, Rajan DK, Ash MR, Muradali D, Simons ME, TerBrugge KG (2003). Efficacy of embolization in traumatic uterine vascular malformations. J Vasc Interv Radiol.

[CR8] Godfrey CD, Zbella EA (2001). Uterine necrosis after uterine artery embolization for leiomyoma. Obstet Gynecol.

[CR9] Hamaguchi S, Okura N, Yoshimatsu M, Ogawa Y, Takizawa K, Nakajima Y (2003). A case of retained placenta increta successfully treated via uterine arterial embolization using N-butyl 2-cyanoacrylate ultrasound. Obstet Gynecol.

[CR10] Horng HC, Hu WM, Tseng HS, Chang WH, Chao KC, Yang MJ (2011). Uterine arterial embolization in the management of severe post-partum hemorrhage: a successful rescue method to avoid peripartum hysterectomy. J Chin Med Assoc.

[CR11] Hulse W, Bahr TM, Morris DS, RichardsDS ISJ, Christensen RD (2020). Emergency-release blood transfusions after postpartum hemorrhage at the Intermountain Healthcare hospitals. Transfusion.

[CR12] Jain K, Fogata M (2007). Retained products of conception mimicking a large endometrial AVM: complete resolution following spontaneous abortion. J Clin Ultrasound.

[CR13] James JL, Chamley LW, Clark AR (2017). Feeding your baby in utero: how the Uteroplacental circulation impacts pregnancy. Physiology.

[CR14] Jansen CHJR, Kastelein AW, Kleinrouweler CE, van Leeuwen E, de Jong KH, Pajkrt E, van Noorden CJF (2020). Development of placental abnormalities in location and anatomy. Acta Obstet Gynecol Scand.

[CR15] Jiang J, Wang C, Xue M (2020). High-intensity focused ultrasound versus uterine artery embolization for patients with retained placenta accreta. Eur J Obstet Gynecol Reprod Biol.

[CR16] Kaufmann P (1985). Basic morphology of the fetal and maternal circuits in the human placenta. Contrib Gynecol Obstet.

[CR17] Kimura Y, Osuga K, Nagai K, Hongyo H, Tanaka K, Ono Y, Higashihara H, Matsuzaki S, Endo M, Kimura T, Tomiyama N (2020). The efficacy of uterine artery embolization with gelatin sponge for retained products of conception with bleeding and future pregnancy outcomes. CVIR Endovasc.

[CR18] Kitahara T, Sato Y, Kakui K, Tatsumi K, Fujiwara H, Konishi I (2011). Management of retained products of conception with marked vascularity. J Obstet Gynaecol Res.

[CR19] Ko HK, Shin JH, Ko GY, Gwon DI, Kim JH, Han K, Lee SW (2017). Efficacy of prophylactic uterine artery embolization before obstetrical procedures with high risk for massive bleeding. Korean J Radiol.

[CR20] Kodan LR, Verschueren KJC, Prüst ZD (2020). Postpartum hemorrhage in Suriname: a national descriptive study of hospital births and an audit of case management. PLoS One.

[CR21] Lee TY, Kim SH, Lee HJ, Kim MJ, Lee SK, Kim YH, Cho SH (2014). Ultrasonographic indications for conservative treatment in pregnancy-related uterine arteriovenous malformations. Acta Radiol.

[CR22] Liu Y, Zhang WW, He M, Gong C, Xie B, Wen X, Li D, Zhang L (2018). Adverse effect analysis of high-intensity focused ultrasound in the treatment of benign uterine diseases. Int J Hyperth.

[CR23] Nyfløt LT, Sandven I, Stray-Pedersen B, Pettersen S, al-Zirqi I, Rosenberg M, Jacobsen AF, Vangen S (2017). Risk factors for severe postpartum hemorrhage: a case-control study. BMC Pregnancy Childbirth.

[CR24] Ohmaru-Nakanishi T, Kuramoto K, Maehara M, Takeuchi R, Oishi H, Ueoka Y (2019). Complications and reproductive outcome after uterine artery embolization for retained products of conception. Obstet Gynaecol Res.

[CR25] Pelage JP, Soyer P, Repiquet D, Herbreteau D, le Dref O, Houdart E, Jacob D, Kardache M, Schurando P, Truc JB, Rymer R (1999). Secondary postpartum hemorrhage: treatment with selective arterial embolization. Radiology.

[CR26] Shams N, Yasmin H, BashirAnswer S, Bai K, Rubab B (2020). Frequency of retained placenta in patients presenting with postpartum haemorrhage after active management of third stage of labour. Int J Community Med Public Health.

[CR27] Sosa CG, Althabe F, Belizán JM, Buekens P (2009). Risk factors for postpartum hemorrhage in vaginal deliveries in a Latin-American population. Obstet Gynecol.

[CR28] Soyer P, Morel O, Fargeaudou Y, Sirol M, Staub F, Boudiaf M, Dahan H, Mebazaa A, Barranger E, le Dref O (2011). Value of pelvic embolization in the management of severe postpartum hemorrhage due to placenta accreta, increta or percreta. Eur J Radiol.

[CR29] Takahashi H, Ohhashi M, Baba Y, Nagayama S, Ogoyama M, Horie K, Suzuki H, Usui R, Ohkuchi A, Matsubara S (2019). Conservative management of retained products of conception in the normal placental position: a retrospective observational study. Eur J Obstet Gynecol Reprod Biol.

[CR30] Takeda A, Koike W (2017). Conservative endovascular management of retained placenta accreta with marked vascularity after abortion or delivery. Arch Gynecol Obstet.

[CR31] Timmerman D, Wauters J, Van Calenbergh S (2003). Color Doppler imaging is a valuable tool for the diagnosis and management of uterine vascular malformations. Ultrasound Obstet Gynecol.

[CR32] Tourne G, Collet F, Seffert P, Veyret C (2003). Place of embolization of the uterine arteries in the management of post-partumhaemorrhage: a study of 12 cases. Eur J Obstet Gynecol Reprod Biol.

[CR33] Vijayakumar A, Srinivas A, Chandrashekar BM, Vijayakumar A (2013). Uterine vascular lesions. Rev Obstet Gynecol.

[CR34] Widmer M, Piaggio G, Hofmeyr GJ, Carroli G, Coomarasamy A, Gallos I, Goudar S, Gülmezoglu AM, Lin SL, Lumbiganon P, Mugerwa K, Owa O, Qureshi Z, Althabe F (2020). Maternal characteristics and causes associated with refractory postpartum haemorrhage after vaginal birth: a secondary analysis of the WHO CHAMPION trial data. BJOG.

